# The New Volume

**Published:** 1858-06

**Authors:** 


### EDITORIAL DEPARTMENT.

*The New Volume.*—Though our Journal is now entering upon the fourteenth year of its existence, the present number shows only the third change in its editorial management, and it can safely be said that it has always been conducted with the principles in view, with which it was instituted. The retiring editor, who has already won as enviable a place among his *confrères* of the daily press, as he had attained by his labors in support of this Journal, has followed the straight path of independent and impartial criticism, which has characterized the entire management.

From the first number, which appeared in June, 1845, and consisted of only twenty pages, to the last number of the thirteenth volume, there is not a single line which lays the editor under the imputation of want of fairness and generosity; and the assurances of hearty support which were then given by the profession, have been more than fulfilled.

With such a retrospective view, the new editor does not feel the emotions of hesitation and diffidence, which otherwise would have been inevitable; he is confident that the hearty coöperation of his professional brethren and the kind consideration of his brother editors, will not be discontinued; and he enters upon his more responsible duties with a hope and confidence, which he has no wish to conceal; not that he expects to equal in own department, the labors of his predecessors, or that he considers himself adequate to the labor, which in times gone by, the editor has been compelled to devote to its pages; but he feels that those days are past, that the Journal is now established, and that the colaborators will make his duties such as he may hope easily to fulfill.

In assuming the entire control, the editor has nothing to ask, but a continuance of the kindly aid, which has contributed so much to the present position of the Journal; and on his part, can only promise to follow the example of his predecessors; with this he extends his warmest greeting to his subscribers, asking their kind indulgence for his labors, and hoping if they should chance to disapprove of any of the views which he may hereafter advance in these pages, that they will impute to him no motives but those of honest conviction, and a sincere desire to advance the true interests of the profession.

